# Distribution and Level of Bioactive Monoacylglycerols in 12 Marine Microalgal Species

**DOI:** 10.3390/md22060258

**Published:** 2024-05-31

**Authors:** Giovanna Santaniello, Gianna Falascina, Marcello Ziaco, Laura Fioretto, Angela Sardo, Martina Carelli, Mariarosaria Conte, Giovanna Romano, Adele Cutignano

**Affiliations:** 1 Stazione Zoologica Anton Dohrn, Ecosustainable Marine Biotechnology Department, via Acton 55, 80133 Naples, Italy; giovanna.santaniello@szn.it (G.S.); angela.sardo@szn.it (A.S.); 2 National Research Council (CNR), Institute of Biomolecular Chemistry (ICB), via Campi Flegrei 34, 80078 Pozzuoli, Italy; giannafalascina96@gmail.com (G.F.); m.ziaco@icb.cnr.it (M.Z.); l.fioretto@icb.cnr.it (L.F.); m.carelli@icb.cnr.it (M.C.); 3 Department of Precision Medicine, University of Campania “Luigi Vanvitelli”, Vico L. De Crecchio 7, 80138 Naples, Italy; mariarosaria.conte@unicampania.it

**Keywords:** microalgae, quantitative MAG analysis, GC-MS, fatty acid analysis, UHPLC-Q-Exactive, untargeted lipid analysis

## Abstract

Microalgae are currently considered an attractive source of highly valuable metabolites potentially exploitable as anticancer agents, nutraceuticals and cosmeceuticals and for bioenergy purposes. Their ease of culturing and their high growth rates further promote their use as raw material for the production of specialty products. In the present paper, we focused our attention on specific glycerol-based lipid compounds, monoacylglycerols (MAGs), which displayed in our previous studies a selective cytotoxic activity against the haematological U-937 and the colon HCT-116 cancer cell lines. Here, we performed a quali/quantitative analysis of MAGs and total fatty acids (FAs) along with a profiling of the main lipid classes in a panel of 12 microalgal species, including diatoms and dinoflagellates. Our results highlight an inter- and intraspecific variability of MAG profile in the selected strains. Among them, *Skeletonema marinoi* (strain FE7) has emerged as the most promising source for possible biotechnological production of MAGs.

## 1. Introduction

The marine environment provides a remarkable reservoir of biological diversity, and organisms from distant phyla are the source of a variety of compounds that can be used and exploited for human needs [1,2,3,4]. Microalgae are one of the most diverse groups of photosynthetic microorganisms present in a wide range of habitats from marine to fresh waters. They encompass at least 800,000 estimated species, of which only 50,000 have been classified [5]. Microalgae-based production of functional and bioactive compounds is of great relevance, considering the environmental advantages of their cultivation at large scale and the possibility of driving and maximising the production of target molecules by tuning cultivation parameters [6]. Lipid yields from microalgae are particularly relevant and, in some species, they can reach values of up to 70% of the total biomass [7,8,9]. Hence, most efforts have been directed towards increasing lipid recovery to obtain, for example, biofuel [10]. On the other hand, microalgae produce metabolites with antibiotic, antifungal, antiviral, anti-inflammatory, antioxidant and anticancer properties, which can be employed as active components or inspire nature-derived molecules for pharmaceutical and nutraceutical applications [11,12,13,14,15,16,17,18,19,20].

Following our ongoing research on microalgal compounds with potential anticancer applications [15,21,22,23,24,25], we recently reported on the occurrence and bioactivity of monoacylglycerols (MAGs) from the marine diatom *Skeletonema marinoi*, which evidenced selective cytotoxic activity against the haematological cancer cell line U-937 and the colon cancer cell line HCT-116, acting as pro-apoptotic agents [26]. This pool of lipids, likely metabolic intermediate of triacylglycerol (TAG) biosynthesis, encompasses several fatty acid derivatives which exhibited different cytotoxic potency, depending on the length and the number of double bonds of the acyl chain [27]. MAGs have been investigated as dietary supplements to deliver beneficial ω-3 fatty acids (FAs). These complex lipids showed a better performance in terms of absorption compared to free ω-3 FAs [28,29], thanks to their stabilising properties. MAGs are also used in the pharmaceutical industry as binders in tablets and as emollients for slow-release drugs [30] and as emulsifiers (E471, E472) in food industry. Although European Food Safety Authority (ESFA) and other authorities have reported on the safety of these additives [31,32,33], there are recent reports suggesting that their assumption could increase the risk of cardiovascular diseases [34] or alter the intestinal microbiota, leading to chronic inflammation [35]. However, the above mentioned classes of emulsifiers are characterised by the presence of a great variety of mono- and diglycerides, and it is still unknown if their detrimental effects are caused by specific features (chain length, unsaturation degrees, association with other compounds). Thus, further studies are needed to better clarify the effect of specific components on human health in order to differentiate molecules with potential side effects from beneficial ones.

## 2. Results

### 2.1. Microalgae Culturing and Extraction

**Figure 1:**
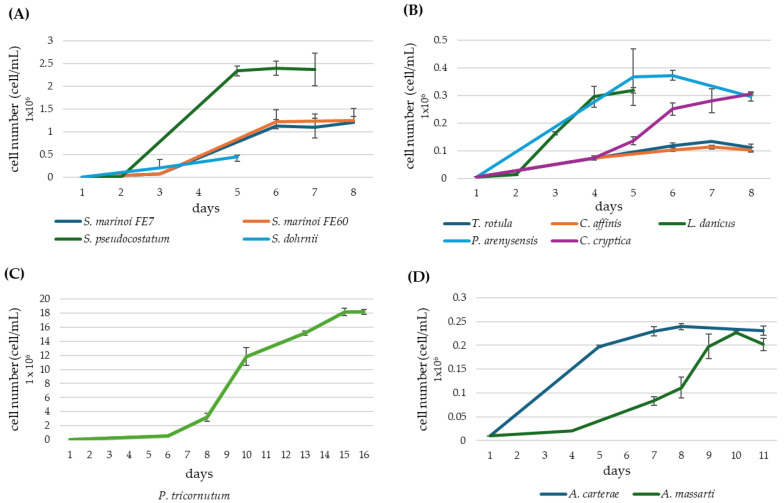
Microalgae growth curves: (**A**) *Skeletonema* genus; (**B**) colonial diatom species plus *Cyclotella cryptica;* (**C**) *Pheodactylum tricornutum*; (**D**) *Amphidinium* genus. Error bars show the standard deviation (SD) of triplicate samples (n = 3).

All the tested diatoms took six to seven days to reach the stationary phase, with the exception of *Phaeodactylum tricornutum*, whose growth curve lasted fifteen days. For dinoflagellates, the plateau was reached in ten to twelve days (Figure 1A–D). The yields of cell pellets obtained from 2 L cultures of each species and lipid yields are reported and normalised in Table 1.

### 2.2. LC-MS Analysis of MAGs

**Figure 2:**
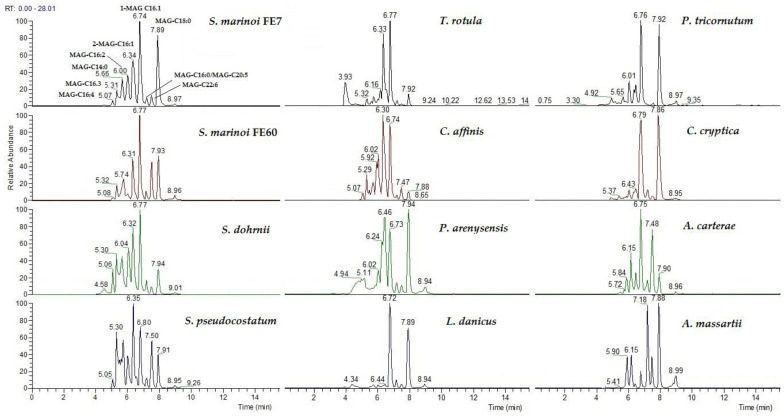
Representative LC-ESIMS profile of MAGs as extracted M + Na^+^ ions in the selected microalgal species.

A preliminary inspection of the qualitative profile of MAGs in the lipid extracts from all the selected microalgal species revealed the occurrence, with various distribution among the different phyla, classes, genera and species, of monoacylglycerols of different fatty acyl lengths with various degrees of unsaturation, namely C14:0, C16 (C16:0, C16:1, C16:2, C16:3 and C16:4.), C18 (C18:0, C18:1, C18:2, C18:3 and C18:4), C20 (C20:0, C20:3, C20:4 and C20:5) and C22 (C22:6) (Figure 2). Starting from this observation, we selected those which appeared to be the most prominent and common species for a quantitative study.

### 2.3. Quantitative LC-MS Analysis of MAGs

In order to perform a quantitative study, pure standards of MAGs were used to prepare calibration curves as reported in Material and Methods by using deuterated MAG-C20:4 as the internal standard (IS). Some of them were commercially available, i.e., MAG-C16:0, MAG-C18:0 and MAG-C18:1. The others were synthesised according to a published procedure [26] as reported in the Supplementary Information (SI-Experimental). All the calibration curve equations gave satisfactory correlation factors (R^2^ ≥ 0.992; Table 2).

**Figure 3:**
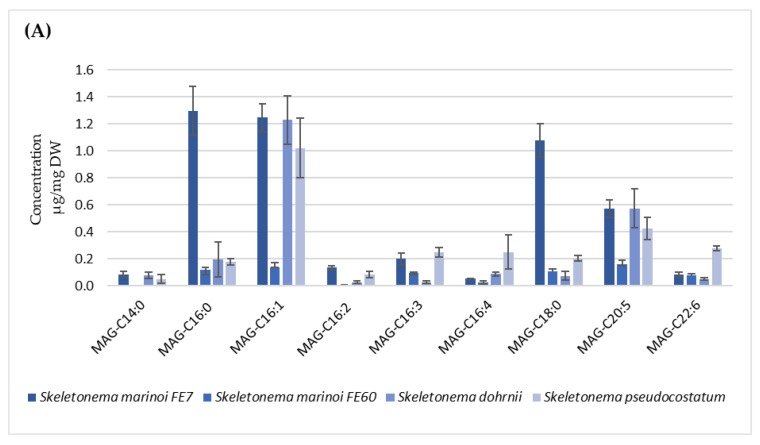

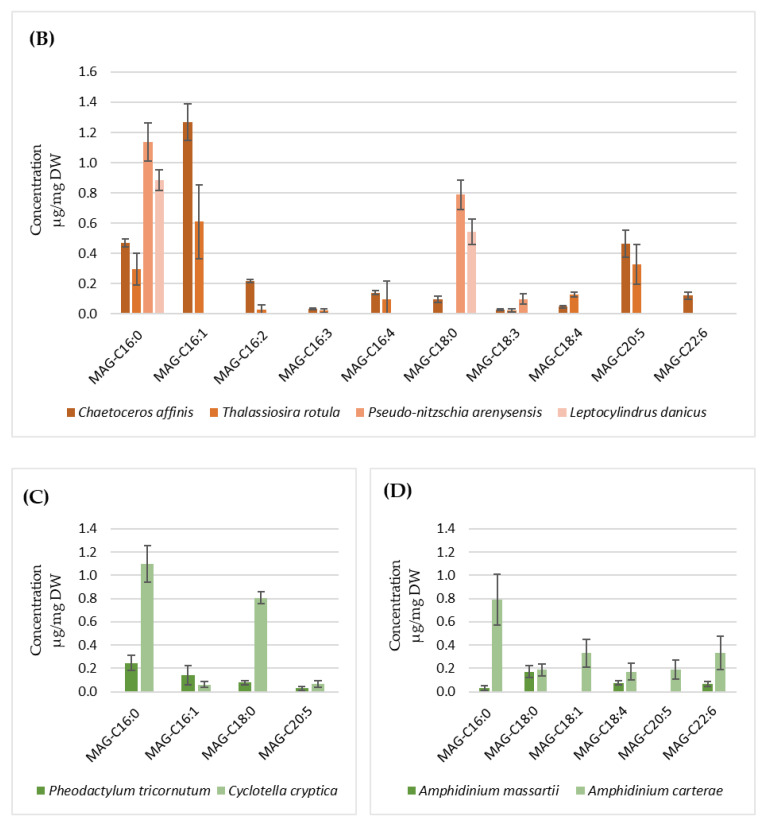
Quantification of main MAGs present in the selected microalgal species. (**A**) *Skeletonema* spp., (**B**) colonial diatoms, (**C**) non-colonial diatoms and (**D**) *Amphidinium* genus. The values of all the molecules are reported as µg/mg of dry pellet. In the figure, we reported molecules whose concentration was higher than 0.02 µg/mg of DW. Results are expressed as mean ± SD (n = 3).

For each species, the amount of MAGs has been expressed both as µg/mg pellet (dry weight, DW, Figure 3A–D and Supplementary Material—Table S1) and as µg/mg lipid extract (Supplementary Material—Table S2). Among all the microalgal species, the best producers of these compounds were those belonging to *Skeletonema* genus (Figure 3A), in particular *S. marinoi* FE7, in which the amount (expressed as µg/mg of DW) of MAG-C16:0, MAG-C16:1, MAG-C18:0 was 1.30, 1.25 and 1.08 µg/mg, respectively. *S. marinoi* FE7, *S. dohrnii* and *S. pseudocostatum* displayed significant production of MAG-C20:5, (0.57, 0.57 and 0.43 µg/mg, respectively), while *S. dohrnii* and *S. pseudocostatum* showed levels of C16:1 comparable to those of *S. marinoi* FE7 (1.23 and 1.02 µg/mg, respectively). *S. pseudocostatum* also yielded an appreciable level of MAG C22:6 (0.28 µg/mg). *T. rotula* appeared to be the poorer producer in terms of absolute amount (Figure 3B), while *L. danicus* and *C. affinis* showed the lower and the higher diversity of MAG species, respectively (Figure 3C). The two dinoflagellates *A. carterae* and *A. massartii*, although not particularly rich in MAGs, were mostly characterized by unsaturated species with C18, C20 and C22 carbon chain length (Figure 3D).

### 2.4. FAME Analysis

The quali/quantitative composition of total fatty acids is reported in Table 3 (as % composition) and in Supplementary Table S3 (as absolute amounts expressed as µg/mg DW). In the selected diatoms, with the exception of *T. rotula* and *P. arenysensis* strains, EPA and palmitoleic acid (C16:1) are the most abundant FAs. All the *Skeletonema* species displayed similar qualitative profiles, EPA and C16:1 accounting for 45–55% of total FAs. With respect to the other microalgal species, which are distinguished by higher contents of saturated FAs (SFA), *Skeletonema* spp. showed a main contribution of polyunsaturated (PUFA) and monounsaturated (MUFA) species, accounting for 80% of total FAs. The only other species that contained a higher percentage of PUFAs was *A. massartii*, where they were represented by C18:4 ω-3 (21.96%), EPA (17.39%) and DHA (21.11%). Namely, *A. massartii*’s levels of DHA were far higher than those of any other species here investigated. Regarding intra-species comparison, no significant difference can be appreciated for the two strains of *S. marinoi*.

### 2.5. Lipid Profiling by LC-MS

A profile of the main lipid classes, including TAGs, glycolipids (GLs) and phospholipids (PLs), is reported in Figure 4 and in Supplementary Materials Figures S1–S8.

**Figure 4:**
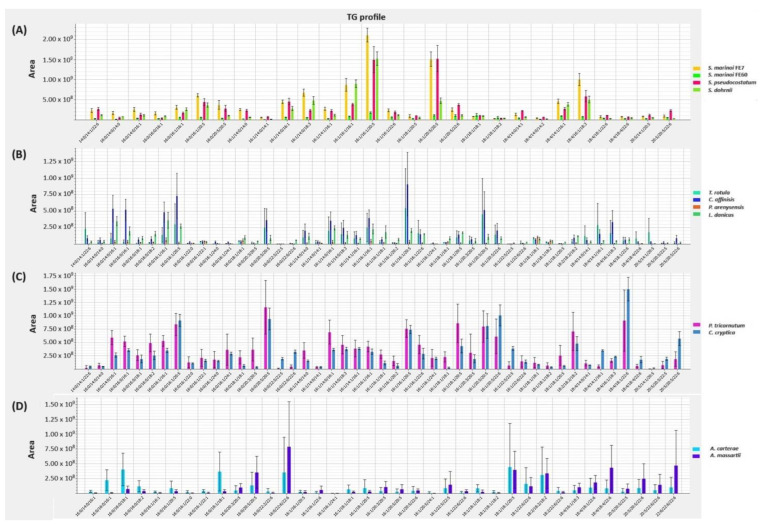
TAG composition in selected microalgal species. Distribution of TAGs in (**A**) four *Skeletonema* spp., (**B)** colonial and (**C**) non-colonial diatoms (**D**) and *Amphidinium* species. Data are reported as mean peak area ± SD (n = 3).

Regardless of the species, the most prominent TAGs in the *Skeletonema* genus were those containing C16:1 and C20:5 FA as shown in Figure 4A, although with different levels based on dry mass normalization.

Among the other diatoms (Figure 4B,C), TAGs in *T. rotula* and *C. affinis* were found to be mainly composed by 14:0, 16:0, 16:1 and EPA; in *P. tricornutum* no specific TAGs species stands out from the profile while in *P. arenysensis* the mostly represented TAGs are composed by saturated and monounsaturated FAs. *L. danicus* exhibited prominently TAGs with a medium chain length (C14–C16). On the other hand, the contribution of highly unsaturated long-chain FAs is remarkable in *C. cryptica* as well as in the two dinoflagellates of the genus *Amphidinium (*Figure 4C,D, respectively).

Compared to TAGs, a limited number of species have been detected for the other lipid classes. Generally, FAs annotated in polar lipids, including PC, PE, PG, DGDG and MGDG, are unsaturated FAs (Supplementary Material, Figures S1–S3, S5 and S6). Conversely, saturated (C14:0 and C16:0) and monounsaturated (C16:1) FAs are dominant in PI and SQDG (Supplementary Material, Figures S4 and S7).

## 3. Discussion

Monoacylglycerols (MAGs) have been recently reported as natural anticancer products isolated from a marine diatom strain, *S. marinoi* FE7 [26]. Their presence has been previously reported in bacteria, fungi, plants and animal tissues [36]. Considering their features, they are largely used in the food industry as emulsifiers and surface-active additives in dairy and bakery products. MAGs have a great potential as dietary supplements, since they can be considered vehicles to deliver the assumption of ω-3 fatty acids, enhancing their absorption and bioavailability compared to other types of formulations based on other lipid derivatives, such as FAs, ethyl esters or triglycerides [28,37].

In view of a possible biotechnological exploitation of microalgae as MAG producers, we aimed at carrying out a targeted lipidomic analysis on twelve microalgal species including diatoms and dinoflagellates, mostly endemic to the Mediterranean Sea, to select the most promising ones to conveniently recover these glycerol-based lipids from natural biomass. The lipid profile of these species, including the previously studied *S. marinoi* FE7, was evaluated and a quantitative analysis was here addressed for the first time by means of UHPLC-HRMS/MS. In order to disclose inter- and intra-genera as well as inter-strain differences, we selected three *Skeletonema* species (*S. marinoi, S. pseudocostatum* and *S. dohrnii*), including two strains of the same species *(S. marinoi* FE7 and FE60), two *Amphidinium* species (*A. carterae* and *A*. *massartii*) and six diatoms belonging to different genera (Table 1 and Section 2.2).

The two diatom species *S. pseudocostaum* and *S. dohrni* showed a MAG profile similar to that of *S. marinoi* FE7, although the proportion of MAG-C16:1 and MAG-C18:0 was reverted. Interestingly, the two strains of *S. marinoi* exhibiting similar growth rates and similar cell concentrations at the stationary phase turned out quite differently in terms of quali/quantitative composition of the various MAGs (Figure 3 and Table S2). In particular, quantitative measurements revealed a lower MAG production in the FE60 strain compared to FE7, suggesting that chemical profile is strain-specific and may vary greatly among taxonomically related species. Previous studies detected a similar intraspecific difference between the same two *S. marinoi* strains for the production of other fatty acid derivatives, oxylipins, suggesting a strain-specific plasticity in secondary metabolite biosynthesis [37].

A comparison of MAG composition among the ten selected diatoms (Supplementary Table S1) highlighted a constant occurrence of MAG-C16:0 in all species, MAG-C18:0 in all but one species and a substantial co-occurrence of MAG-C16:1 and MAG-C20:5. The two dinoflagellates exhibited a poorer variety and lower level of MAG. However, it is worth mentioning that the highest level of MAG-C22:6 was found in *A. carterae* (0.33 µg/mg), while it was undetectable or present in trace amounts in the other species, with the only exception being *S. pseudocostatum* (0.28 µg/mg). From the perspective of a large-scale production of these compounds, the production of MAGs is most promising in terms of abundance (>1 µg/mg DW for the main species) and variety in *S. marinoi* FE7, also considering the growth performance and the yield in term of grams of dry weight per litre of culture (Table 1 and Supplementary Table S1).

FAs annotated in MAGs showed a substantial overlap with total FA composition, although it is worth noting the unusually high proportion of stearic acid (C18:0) in MAGs compared with its minor occurrence in total FAs.

Natural 2-MAG usually derives from lipase-mediated hydrolysis of TAGs; an alternative source is polar phospholipids [30]. In order to elucidate the biosynthetic origin of microalgal MAGs, we performed a lipidomic analysis, focusing on the main glycerolipid classes, i.e., TAGs, SQDG, MGDG, DGDG, PE, PI, PG and PC. C16:0, C16:1 are the most abundant FAs in all lipid species while C20:5 occurs in TAGs, PC, PE, PG and glycoglycerolipids. Apart from minor SQDG species, i.e., SQDG 16:0_18:0, SQDG 18:0_18:3, SQDG 18:0_20:5 (Supplementary Material Figure S8), the occurrence of C18:0 was not annotated in any other lipid species. This could be due to the presence of a SQDG specific lipase; although it does not seem that the three SQDG species containing C18:0 are specific to the microalgae where MAG-C18:0 has been mainly detected (Supplementary Material Table S1 and Figure S8). Hence, the origin of this MAG remains uncertain, and further investigations are required to clarify the source of this bioactive lipid and if its synthesis could be modulated by modifying culture conditions in terms of physico-chemical parameters.

Several MAG species are recognized as signalling lipid molecules in different tissues in higher organisms: the endocannabinoid 2-arachidonoylglycerol is the most studied MAG, and its activity has been associated with many physiological processes, including inflammation, food intake, learning and memory and epileptogenesis [38,39,40]. Recent studies have shown that 1-MAGs, especially 1-MAGs of saturated fatty acids (MAG-C16:0 and MAG-C18:0), are also signalling molecules, as they are involved in the regulation of insulin secretion and insulin sensitivity and also in adipose browning via peroxisomal proliferator-activated receptor PPARα and PPARγ activation [41,42,43]. Moreover, 1-MAG with the unsaturated fatty acid DHA showed antihypertensive properties in rat models and reduced the levels of pro-inflammatory markers, such as CRP, IL-6, TNFα and IL-1β [44,45].

## 4. Materials and Methods

### 4.1. General Techniques and Chemicals

NMR spectra were recorded on a Bruker Avance DRX 600 (Bruker, Milan, Italy) operating at 600 MHz for protons, equipped with an inverse TCI CryoProbe fitted with a gradient along the Z-axis or on an Avance III HD operating at 400 MHz for protons, equipped with a CryoProbe Prodigy. Chemical shift values are reported in ppm (δ) and referenced to internal signals of residual protons (for CDCl_3_ ^1^H δ 7.26, ^13^C 77.0 ppm). LC-MS analyses were performed on a Q-Exactive Hybrid Quadrupole-Orbitrap mass spectrometer (Thermo Scientific, San Jose, CA, USA) equipped with a HESI source and coupled with an Infinity 1290 UHPLC System (Agilent Technologies, Santa Clara, CA, USA) on a Kinetex Biphenyl column, 2.6 µm, 150 × 2.1 mm (Phenomenex, Castel Maggiore, Bologna, Italy). GC-MS analysis was carried out on ion-trap mass spectrometer operating in EI mode (70 eV) (Thermo-Scientific, Polaris Q) connected to a gas chromatographic system (Thermo-Scientific, GCQ) equipped with a 5% phenyl/methyl polysiloxane column (30 m × 0.25 mm × 0.25 µm, Agilent, VF-5ms) using high-purity helium as the gas carrier. HPLC purification of synthetic MAGs was performed on a Shimadzu high-performance liquid chromatography system using a Shimadzu liquid chromatograph (Shimadzu, Kyoto, Japan) LC-20ADXR equipped with a Diode Array Detector SPDM-20A and a Synergi-Fusion RP column 80A, 150 × 4.6 mm, 4 µm (Phenomenex). Tricosanoic acid, Marine PUFA-3 standard, MTBE (HPLC grade), MeOH (HPLC/LC-MS grade), petroleum ether and diethyl ether (analytical grade) were all purchased from Merck (Milan, Italy). 1-Arachidonoyl glycerol-*d*8 (MAG-C20:4-*d*8, IS), 1-oleoyl glycerol (MAG-C18:1), 1-linoleoyl glycerol (MAG-C18:2), 1-palmitoyl glycerol (MAG-C16:0), 1-stearoyl glycerol (MAG-C18:0), (5*Z*,8*Z*,11*Z*,14*Z*,17*Z*)-eicosa-(5,8,11,14,17)-pentaenoic acid (EPA), arachidonic acid (ARA), (4*Z*,7*Z*,10*Z*,13*Z*,16*Z*,19*Z*)-docosa-4,7,10,13,16,19-hexaenoic acid (DHA) and (6*Z*,9*Z*,12*Z*)-Hexadeca-6,9,12-trienoic acid (C16:3) were obtained from Cayman Chemical (Vinci Biochem, Vinci, FI, Italy). Water for LC MS was obtained using a MilliQ Apparatus (Millipore, Milan, Italy). TLC plates (KieselGel 60 F254) and silica gel powder (KieselGel 60, 0.063–0.200 mm) were from Merck (Milan, Italy).

### 4.2. Biological Material

The strains *Skeletonema marinoi* FE7, *S. marinoi* FE60, *Skeletonema dohrnii* FE82, *Skeletonema pseudocostatum* BS4, *Thalassiosira rotula* FE80, *Pseudo-nitzschia arenysensis* MC1248-12, *Leptocylindrus danicus* Na74A2 and *Chaetoceros affinis* Na57B2 were available from the Stazione Zoologica Anton Dohrn and are included in the culture collection of the Department of Ecosustainable Marine Biotechnology. With the exception of the strain FE7 and FE60, isolated from a seawater sample collected from the Adriatic Sea, the other species were isolated by the personnel of the Stazione Zoologica from the Gulf of Naples via the capillary pipette method, i.e., by picking up single cells from seawater samples using glass pipettes with fine capillary tips [46]. Endemic diatom species were identified through PCR amplification, sequencing and alignment of the nuclear-encoded large subunit (LSU) rDNA for species belonging to the *Skeletonema* genus, for *C. affinis* and *T. rotula* [47]; internal transcribed spacer 2 (ITS2) for *Pseudo-nitzschia arenysensis* [48]; small subunit (SSU) rDNA for *L. danicus* [49]. *Phaeodactylum tricornutum* CCMP632, *Cyclotella cryptica* CCMP332, *Amphidinium carterae* CCMP 121 and *Amphidinium massartii* ARC149 are commercial strains purchased from the Provasoli-Guillard National Center for Marine Algae and Microbiota (NCMA, 60 Bigelow Drive East Boothbay, ME 04544) or the Algal Resources Collection (ARC, 5600 Marvin K. Moss Lane, Wilmington NC, 28409, USA) culture collections.

### 4.3. Microalgae Culturing

Diatoms were grown in f/2 medium [50] and dinoflagellates in K medium [51] in 2 L polycarbonate bottles, under constant bubbling (air flow 3.5 L/min) provided by fill-venting devices. Air flow was filtered through 0.22 µm membrane filters to ensure sterility. Each experiment was performed in triplicate.

Microalgae (starting cell density: 5000 cell/mL) were maintained in a climate chamber at 18 °C, with a 12:12 h light:dark regime, under a light intensity of 100 µmol photons m^−2^ s^−1^. Cell concentration was estimated daily using Bürker (Eberstadt, Germany) and Sedgewick–Rafter counting chambers (KC, Silkeborg, Denmark) under an optical microscope (Axiovert ZEISS 200, Obertkochen, Germany) to obtain growth curves. Cultures were harvested at the onset of the stationary phase by centrifugation (Biofuge Fresco Heraeus, Cavenago di Brianza, Monza and Brianza, Italy) at 3600 rpm, 10–15 min, at 4 °C. Wet pellets were immediately frozen in liquid nitrogen and stored at −80 °C.

### 4.4. Microalgal Cell Pellet Extraction

Frozen wet pellets of all strains were freeze-dried with a Modulyo EF4 (Edwards, Cinisello Balsamo, Milan, Italy). Two aliquots of the dry biomass (10 mg) from each replicate were extracted following the MTBE/MeOH protocol as previously reported [52]. Briefly, solvent extraction was performed by adding to the samples 300 µL MeOH, 1 mL of MTBE and 400 ng of 1-Arachidonoyl Glycerol-*_d8_* as the IS. Phase separation was induced by adding 250 µL of MilliQ water. After centrifugation at 10,000× *g* at 4 °C for 10 min (microcentrifuge MicroStar 17R, VWR International, Milan, Italy), the organic phase was collected and transferred into a pre-weighed vial. The remaining aqueous phase was re-extracted with 350 µL of MTBE, and the extracts were combined. After removal of the organic solvent under a stream of nitrogen, the extracts were further dried under vacuum, weighted and stored at −20 °C until the analysis.

### 4.5. Quantitative Analysis of MAGs

Commercial and in-house synthesized MAG standards were used to prepare the calibration curves with at least five calibration points in the range of 10–10,000 ng/mL and IS (MAG-C20:4-*d*8) 400 ng/mL. Each curve was performed in triplicate.

LC-MS analysis was achieved on a Q-Exactive platform according to Miceli et al. [26]. Briefly, the column was a Kinetex Biphenyl column, 2.6 µm, 150 × 2.1 mm (Phenomenex, Castel Maggiore, Bologna, Italy) kept at 28 °C. Eluent A: water; eluent B: MeOH. The elution program consisted of a gradient from 40% to 80% B over 2 min, then to 100% B over 13 min, holding at 100% B for 7 min. The flow rate was 0.3 mL/min. Full MS scans were acquired in positive polarity over the range *m*/*z* 200–1200. A targeted MS/MS method was used for molecular ion confirmation. A stepped normalised energy of 25–28–35% was applied. The injection volume was 10 µL.

Calibration curves were plotted considering as response the area ratio of each MAG standard/IS *vs* MAG concentration. The peak area was measured on the extracted ion chromatogram of the molecular ion [M + Na]^+^ after its confirmation by MS/MS. A least-square linear regression weighting by the reciprocal of the concentration was used to best fit the linearity curve.

Quantitative processing of raw data was performed in the Thermo Xcalibur quan browser (Thermo Fisher Scientific Inc., version 2.2 SP1.48, Waltham, MA, USA).

MAG-C16:0, MAG-C16:3, MAG-C18:0, MAG-C18:1, MAG-C18:2, MAG-ARA, MAG-EPA and MAG-DHA were quantified using the respective calibration curves constructed on pure commercial/synthetic standards.

MAG-C14:0, MAG-C16:1, MAG-C16:2, MAG-C16:4, MAG-C18:3, MAG-C18:4, MAG-C20:0 and MAG-C20:3 were measured using the calibration curves constructed on the MAG standards containing the same number of double bonds (i.e., the calibration curve of MAG-C18:1 was used to infer MAG-16:1 concentration). The amounts of MAGs are reported as µg/mg of dry material or as µg/mg of lipid extract.

### 4.6. Total Fatty Acid Analysis by GC-MS

Total lipids were extracted from the dry pellet (5 mg) of each microalgal sample in triplicate as described above, adding 40 µg of C23:0 fatty acid methyl ester as IS. Methanolysis was applied to convert bound FAs into their corresponding methyl ester derivatives (FAME): lipid extracts were dissolved in 500 µL of methanol, and a spatula tip of sodium carbonate (Na_2_CO_3_) was added. The mixture was left to react overnight at 45 °C. The reaction mixture was then diluted with MilliQ water, and the basic solution neutralised with hydrochloric acid (HCl) 6 M. The aqueous reaction mixture was subsequently extracted twice with diethyl ether; the organic phase was transferred into pre-weighed vials and dried under N_2_ stream and under vacuum to remove solvent traces. Free FAs were converted into their corresponding methyl esters (FAME) by adding an excess (1 mL) of an ethereal solution of diazomethane (CH_2_N_2_) freshly prepared in-house from Diazald^®^ (Merck, Milan, Italy) using a commercial glass kit purchased by Merck [53]. After a reaction time of 60 min, samples were dried under N_2_ stream and stored at −20 °C. For GC-MS analysis, the samples were dissolved in dichloromethane (300 µL) and transferred into autosampler vials. The following temperature gradient was applied: initial 160 °C holding for 3 min; then 5 °C/min up to 260 °C, followed by 30 °C/min up to 310 °C, holding for 3 min at 310 °C; split flow 10 mL/min; full scan *m*/*z* 50–450. Injection volume: 2 µL. Analytical runs were processed using Xcalibur software (Thermo Fisher Scientific Inc., version 1.4 SR1). FAME peaks were identified by comparing their elution times and MS spectra with a commercial standard pool (Marine PUFA-3, 1 mg mL^−1^) and the NIST database. For quantitative measurement, the peak area of each FAME (x) was normalized by IS and expressed as µg/mg dry material as follows:

FAME (µg/mg_DWn_) = (A_x_ × 40)/(A_IS_ × mg_DW_)

### 4.7. Untargeted Lipidomics by UHPLC-HRESIMS

Total lipids were extracted from all microalgal species (10 mg of dry biomass) in biological triplicate as reported in Paragraph 4.4 and analysed in technical replicate according to the method reported in Cutignano et al. [23]. Raw data were processed with LipidSearch software (Thermo-Scientific, version 4.1) for lipid identification and manually checked to confirm both the annotation and the integration of ion peak areas. Data were plotted as peak areas of the following extracted molecular ions: TAGs, DGDG, MGDG and PC as [M + Na]^+^; SQDG, PE, PG and PI as [M−H^−^].

## 5. Conclusions

Twelve marine microalgal species were screened for the production of MAGs exploitable as anticancer compounds or as nutraceuticals. The quali/quantitative analysis showed a relatively high inter- and intraspecific variability in MAG profiles, with a predominant presence of MAG-C16:0, MAG-C18:0 and/or MAG-C16:1 in the species with the highest MAG production levels, which include *S. marinoi* FE7, *S. pseudocostatum*, *S. dohrnii*, *P. arenysensis*, *C. cryptica*, *C. affinis* and *A. carterae*. Above all, *S.marinoi* FE7, for chemical and culturing features, distinguished as the most promising natural source for a biotechnological production of bioactive MAGs. Further investigations are needed to disclose the biosynthetic origin of this group of lipids.

## Figures and Tables

**Table 1:** List of the cultivated species and related yields of the harvested biomass expressed as grams (g) of fresh weight (FW), of dry weight (DW) and the corresponding yields of the lipid fraction expressed as mg/g of DW. Results are expresses as mean ± SD (n = 3).

Microalgal Species	Biomass Yield (g FW/L)	Biomass Yield (g DW/L)	Lipid Yield (mg/g DW)
*Skeletonema marinoi* FE7	0.61 ± 0.03	0.13 ± 0.01	124.47 ± 33.32
*Skeletonema marinoi* FE60	0.70 ± 0.05	0.17 ± 0.01	112.60 ± 6.33
*Skeletonema dohrnii*	0.52 ± 0.02	0.10 ± 0.01	110.33 ± 6.98
*Skeletonema pseudocostatum*	0.63 ± 0.04	0.14 ± 0.01	142.85 ± 21.01
*Chaetoceros affinis*	0.97 ± 0.06	0.10 ± 0.01	171.14 ± 14.03
*Thalassiosira rotula*	0.59 ± 0.02	0.10 ± 0.00	261.18 ± 28.50
*Pseudo-nitzschia arenysensis*	0.31 ± 0.02	0.03 ± 0.00	121.04 ± 12.41
*Leptocylindrus danicus*	0.41 ± 0.01	0.05 ± 0.00	170.40 ± 7.28
*Cyclotella cryptica*	0.24 ± 0.02	0.05 ± 0.00	223.48 ± 7.18
*Phaeodactylum tricornutum*	1.52 ± 0.12	0.34 ± 0.02	177.53 ± 39.31
*Amphidinium carterae*	0.66 ± 0.06	0.12 ± 0.01	200.71 ± 18.55
*Amphidinium massartii*	0.49 ± 0.09	0.09 ± 0.02	226.89 ± 25.60

**Table 2:** Calibration curves obtained with commercial and synthetic MAG standards.

Selected MAGs	Calibration Curve Equation	R^2^
MAG-C16:0	y = 0.376283 + 2.66559x	0.9923
MAG-C16:3	y = 0.0336387 + 3.44269x	0.9926
MAG-C18:0	y = 1.35265 + 3.62941x	0.9930
MAG-C18:1	y = 1.13375 + 2.03456x	0.9926
MAG-C18:2	y = 0.495324 + 3.39005x	0.9973
MAG-C20:4	y = 0.204654 + 4.369x	0.9944
MAG-C20:5	y = 0.458159 + 2.94969x	0.9919
MAG-C22:6	y = 1.0052 + 4.21918x	0.9942

**Table 3:** FA composition in the selected microalgal species. Values are expressed as relative abundance on total amount (mean % ± SD, n = 3) based on GC-MS peak areas. FA percentages > 10% are highlighted in bold.

		Diatoms										Dinoflagellates	
FAME	Rt	*S. marinoi* FE7	*S. marinoi* FE60	*S. dohrnii*	*S. pseudocostatum*	*T. rotula*	*C. affinis*	*P. tricornutum*	*P. arenysensis*	*L. danicus*	*C. cryptica*	*A. carterae*	*A. massartii*
**C14:0**	8.52	8.25 ± 0.25	8.42 ± 0.39	8.07 ± 0.15	**15.21 ± 0.36**	**26.35 ± 0.78**	**15.19 ± 0.73**	3.85 ± 0.08	**11.82 ± 0.14**	**12.15 ± 0.44**	2.89 ± 0.07	1.76 ± 0.33	
**C15:0**	10.85	0.45 ± 0.03	0.30 ± 0.01	0.47 ± 0.01	0.59 ± 0.02	0.76 ± 0.03	0.67 ± 0.04	0.17 ± 0.03	0.71 ± 0.13	0.51 ± 0.09	0.66 ± 0.02	0.12 ± 0.01	
**C16:4 ω1**	12.46	4.19 ± 0.13	5.97 ± 0.23	5.22 ± 0.15	9.29 ± 0.08	1.44 ± 0.32	2.23 ± 0.50	1.67 ± 0.15	4.51 ± 0.46	4.28 ± 0.40			
**C16:3 ω4**	12.62	8.87 ± 0.37	5.85 ± 0.08	**10.33** ± 0.07	7.75 ± 0.02	0.86 ± 0.11	0.69 ± 0.07	2.69 ± 0.29	5.89 ± 0.20	1.15 ± 0.14	**13.86** ± 0.31		
**C16:1 ω7**	12.93	**33.63 ± 0.39**	**33.11 ± 1.66**	**28.77 ± 0.32**	**23.10 ± 0.23**	**40.68 ± 0.98**	**38.62 ± 1.69**	**34.37 ± 0.33**	**31.92 ± 0.24**	**29.40 ± 0.89**	**31.51 ± 0.11**	0.37 ± 0.03	0.03 ± 0.02
**C16:2 ω3**	13.01	4.98 ± 0.12	5.98 ± 0.08	4.91 ± 0.37	3.51 ± 0.07	1.43 ± 0.09	4.45 ± 0.25	1.64 ± 0.18	5.18 ± 0.26	8.25 ± 0.65	3.21 ± 0.04		
**C16:0**	13.45	6.74 ± 0.17	6.64 ± 0.27	7.61 ± 0.17	5.08 ± 0.10	**11.24 ± 0.51**	**18.06 ± 0.83**	**18.56 ± 0.41**	7.64 ± 0.69	9.80 ± 0.34	**18.44 ± 0.07**	**33.20 ± 1.90**	**20.12 ± 0.40**
**C18:3 ω6**	17.46	0.22 ± 0.03	0.19 ± 0.02	0.27 ± 0.01	0.43 ± 0.00					0.18 ± 0.02	0.14 ± 0.02		
**C18:3 ω3**	17.56	0.23 ± 0.05	0.44 ± 0.05		0.11 ± 0.02	1.56 ± 0.07	0.45 ± 0.10	0.54 ± 0.03	0.38 ± 0.00	0.49 ± 0.05	0.14 ± 0.02	0.13 ± 0.01	0.07 ± 0.07
**C18:5 ω3 + C18:4 ω3**	17.75	3.88 ± 0.04	2.21 ± 0.15	3.48 ± 0.04	1.35 ± 0.06	2.09 ± 0.45	0.48 ± 0.10	1.38 ± 0.09	0.74 ± 0.07	0.74 ± 0.13	3.90 ± 0.07	**16.55 ± 1.52**	**21.90 ± 0.39**
**C18:2 ω6**	18.00	0.77 ± 0.03	1.23 ± 0.04	1.24 ± 0.01	0.62 ± 0.02	0.75 ± 0.03	0.32 ± 0.02	2.34 ± 0.14	1.89 ± 0.03	4.63 ± 0.49	0.19 ± 0.02	1.14 ± 0.03	0.45 ± 0.06
**C18:1 ω9**	18.19	0.39 ± 0.00	0.78 ± 0.05	0.73 ± 0.02	1.78 ± 0.01	2.73 ± 0.03	0.82 ± 0.09	3.60 ± 0.02	5.59 ± 0.41	4.97 ± 1.35	0.22 ± 0.00	9.31 ± 2.24	1.20 ± 0.03
**C18:1 ω7**	18.32	1.18 ± 0.16	1.23 ± 0.05	1.76 ± 0.04	0.26 ± 0.02	1.99 ± 0.11	2.36 ± 0.11	0.54 ± 0.00	1.59 ± 0.24	0.78 ± 0.09	0.58 ± 0.03	1.22 ± 0.42	0.01 ± 0.01
**C18:2 ωx**	18.50	1.35 ± 0.26	0.64 ± 0.27	2.28 ± 0.29	3.21 ± 0.06	2.25 ± 0.03	2.03 ± 0.13	0.42 ± 0.07	4.22 ± 0.33	4.63 ± 0.49	2.00 ± 0.05	5.35 ± 0.71	3.86 ± 0.20
**C18:0**	18.87	0.61 ± 0.09	0.85 ± 0.16	0.60 ± 0.05	0.60 ± 0.00	0.72 ± 0.02	1.03 ± 0.03	0.62 ± 0.05	0.91 ± 0.11	0.87 ± 0.10	0.53 ± 0.04	3.20 ± 0.08	3.60 ± 0.03
**AA**	22.45		0.10 ± 0.01			0.11 ± 0.03	0.05 ± 0.01	0.35 ± 0.04	0.22 ± 0.05	0.41 ± 0.20			0.48 ± 0.03
**EPA**	22.65	**21.43 ± 0.37**	**22.92 ± 1.78**	**20.91 ± 0.30**	**23.66 ± 0.43**	4.63 ± 1.02	**11.76 ± 2.61**	**25.82 ± 0.37**	9.10 ± 1.97	**13.57 ± 1.29**	**19.11 ± 0.17**	**10.60 ± 0.86**	**17.36 ± 0.18**
**C21:0**	24.30					0.07 ± 0.01			0.37 ± 0.05			5.55 ± 0.21	6.96 ± 0.11
**DHA**	27.51	2.83 ± 0.04	3.12 ± 0.36	3.35 ± 0.08	3.45 ± 0.12	0.31 ± 0.10	0.53 ± 0.16	1.35 ± 0.02	0.51 ± 0.15	2.89 ± 0.21	2.58 ± 0.07	7.91 ± 1.01	**21.18 ± 0.66**
**C22:0**	29.50					0.04 ± 0.00		0.09 ± 0.00	1.76 ± 0.31			1.16 ± 0.05	1.82 ± 0.04
**C24:0**	31.91						0.26 ± 0.02	2.26 ± 0.08	5.04 ± 0.85		0.02 ± 0.00	2.42 ± 0.11	0.95 ± 0.04
**SFA**		16.05 ± 0.21	16.31 ± 0.73	16.75 ± 0.11	21.50 ± 0.44	39.15 ± 1.16	35.27 ± 1.61	25.59 ± 0.14	28.24 ± 2.16	23.62 ± 0.68	22.54 ± 0.02	47.41 ± 1.81	33.45 ± 0.29
**MUFA**		35.19 ± 0.51	35.21 ± 1.67	31.26 ± 0.30	25.15 ± 0.22	45.44 ± 1.10	41.87 ± 1.88	38.23 ± 0.10	39.10 ± 0.65	35.15 ± 1.52	32.32 ± 0.09	10.90 ± 1.87	1.24 ± 0.05
**PUFA**		48.75 ± 0.59	48.48 ± 2.26	51.99 ± 0.38	53.35 ± 0.66	15.40 ± 2.03	22.87 ± 3.49	37.48 ± 0.71	32.66 ± 2.41	41.23 ± 2.20	45.14 ± 0.07	41.69 ± 3.68	65.29 ± 0.32

## Data Availability

The data presented in this study are available on request from the corresponding authors.

## Supplementary Materials

The following supporting information can be downloaded at https://www.mdpi.com/article/10.3390/md22060258/s1, Supplementary methods. Preparation of synthetic MAGs; Figure S1. PC composition in the selected microalgal species. Data are reported as mean peak area ± SD (n = 3).; Figure S2. PE composition in the selected microalgal species. Data are reported as mean peak area ± SD (n = 3); Figure S3. PG composition in the selected microalgal species. Distribution of PG in (A) four *Skeletonema* spp. (B) other colonial and (C) non-colonial diatoms (D) and the two *Amphidinium* species. Data are reported as mean peak area ± SD (n = 3).; Figure S4. PI composition in the selected microalgal species. Distribution of PI in (A) four *Skeletonema* spp. (B) other colonial and (C) non-colonial diatoms (D) and the two *Amphidinium* species. Data are reported as mean peak area ± SD (n = 3); Figure S5. DGDG composition in the selected microalgal species. Distribution of DGDG in (A) four *Skeletonema* spp. (B) other colonial and (C) non-colonial diatoms (D) and the two *Amphidinium* species. Data are reported as mean peak area ± SD (n = 3); Figure S6. MGDG composition in the selected microalgal species. Distribution of MGDG in (A) four *Skeletonema* spp. (B) other colonial and (C) non-colonial diatoms (D) and the two *Amphidinium* species. Data are reported as mean peak area ± SD (n = 3); Figure S7. SQDG composition in the selected microalgal species. Distribution of SQDG in (A) four *Skeletonema* spp. (B,C) colonial and (D) non-colonial diatoms (E) and the two *Amphidinium* species. Data are reported as mean peak area ± SD (n = 3); Figure S8. SQDG containing C18:0 fatty acid in selected microalgal species. Data are reported as mean peak area ± SD (n = 3). Table S1. Abundance of main MAGs quantified by LC-MS analysis and expressed as µg/mg of DW. Means ± SD (n = 3); Table S2. Abundance of main MAGs quantified by LC-MS analysis and expressed as µg/mg lipid extract. Means ± SD (n = 3); Table S3. Levels of main fatty acids identified and quantified by GC-MS analysis as methylester derivatives (FAMEs). Values are expressed as µg/mg DW. Means ± SD (n = 3).
