# Supplementary material for: Distribution and Level of Bioactive Monoacylglycerols in 12 Marine Microalgal Species
Source: Mar Drugs. 2024 May 31;22(6):258. doi: 10.3390/md22060258 (PMC11205161; doi:10.3390/md22060258)
Supplement: Supplementary file 1 [file marinedrugs-22-00258-s001.zip › marinedrugs-3014189-supplementary.pdf]

Supplementary Material

Distribution and level of bioactive monoacylglycerols in 12 marine microalgal species

Giovanna Santaniello[1,2,3], Gianna Falascina,[2] Marcello Ziaco[2], Laura Fioretto[2], Angela Sardo[1], Martina Carelli [2], Mariarosaria Conte[3], Giovanna Romano[1*] and Adele Cutignano[1,2,*]

1 Stazione Zoologica Anton Dohrn - Ecosustainable Marine Biotechnology Department, via Acton 55, 80133 Naples, Italy;
2 National Research Council (CNR) – Institute of Biomolecular Chemistry (ICB), via Campi Flegrei 34, 80078 Pozzuoli (Naples), Italy;
3 Department of Precision Medicine, University of Campania "Luigi Vanvitelli", Vico L. De Crecchio 7, 80138 Naples, Italy;

*Corresponding authors: acutignano@icb.cnr.it (A.C); giovanna.romano@szn.it (G.R)

Supplementary Methods

Preparation of synthetic MAG

Synthesis of MAGs was carried out from 1.2-O-isopropylidene glycerol and the desired fatty acid as previously reported (Miceli et al.. 2019). Details are indicated below.

1.2-O-isopropylidene glycerol: Glycerol (1.0 g. 0.012 mol) was dissolved in anhydrous N.N-dimethylformamide (2 mL); 2.2-dimethoxypropane (2 mL) and p-toluenesulfonic acid (150 mg) were added; after stirring overnight at room temperature. the mixture was partitioned between water and dichloromethane; the organic phase was purified by silica gel chromatography using a gradient of petroleum ether/diethyl ether to give 1.2-O-isopropylidene glycerol (1.5 g. 0.011 mol. 68%).

^{1}H-NMR ($CDCl_3$): δ 4.24 (1H, m, H-2), 4.09 (1H, dd, J = 6.7, 8.5 Hz, H-1a), 3.82 (1H, dd, J = 6.4, 8.5 Hz, H-1b), 3.65 (2H, m, H_2-1), 1.46 (3H, s, CH3), 1.40 (3H, s, CH_3).

- 1-MAG-ARA

1.2-O-isopropylidene-3-O-arachidonoylglycerol: 1.2-O-isopropylidene glycerol (0.325 g. 0.00077 mol) was dissolved in anhydrous dichloromethane (5 mL); arachidonic acid (0.279 g. 0.00092 mol). N.N-dicyclohexylcarbodiimide (0.188 g. 0.00092 mol). and N.N-dimethylaminopyridine (0.006 g. 0.00005 mol) were added under argon and the reaction mixture was stirred overnight at room temperature; after evaporation under reduced pressure. the mixture was purified by silica gel chromatography using a gradient of petroleum ether/diethyl ether to give 1.2-O-isopropylidene-3-O-arachidonoylglycerol (0.301 g. 0.00072 mol. 93%).

1-mono-O-arachidonoylglycerol: 1.2-O-isopropylidene-3-O-arachidonoylglycerol (0.301 g. 0.00072 mol) was dissolved in methanol/chloroform/water mixture (3/3/1) (7 mL) and Dowex H+ resin (8 g) was added; the mixture was stirred at room temperature for 3 h. After filtration. the filtrate was purified by silica gel chromatography using a gradient of petroleum ether/diethyl ether to give the mono-O-arachidonoylglycerol (0.15 g. 0.00040 mol. 56%). ^{1}H-NMR (600 MHz; $CD_3OD/CDCl_3$ 1/1): δ 5.37-5.31 (8H, vinyl protons), 4.13 (1H, dd, J = 4.4, 11.2 Hz), 4.07 (1H, dd, J = 6.0, 11.2 Hz), 3.83 (1H, m), 3.58 (1H, dd, J = 4.8, 11.2 Hz), 3.53 (1H, dd, J = 5.9, 11.2 Hz), 2.86–2.71 (6H, m), 2.35 (2H, t, J = 7.7 Hz, α-

methylene of arachidonoyl portion), 2.16–2.01 (4H, allylic protons), 1.68 (2H, m, β-methylene of arachidonoyl portion), 1.36–1.24 (aliphatic protons), 0.86 (3H, t, *J* = 6.68 Hz). HR-ESIMS *m*/*z* 401.2664 $[M + Na]^+$.

- 1-MAG-EPA

1.2-O-isopropylidene-3-O-eicosapentaenoylglycerol: 1.2-O-isopropylidene glycerol (0.325 g. 0.00077 mol) was dissolved in anhydrous dichloromethane (5 mL); eicosapentaenoic acid (0.232 g. 0.00092 mol). N.N-dicyclohexylcarbodiimide (0.188 g. 0.00092 mol). and N.N-dimethylaminopyridine (0.006 g. 0.00005 mol) were added under argon and the reaction mixture was stirred overnight at room temperature; after evaporation under reduced pressure. the mixture was purified by silica gel chromatography using a gradient of petroleum ether/diethyl ether to give 1.2-O-isopropylidene-3-O-eicosapentaenoylglycerol (0.298 g. 0.00069 mol. 90%).

1-mono-O-eicosapentaenoylglycerol: 1.2-O-isopropylidene-3-O-eicosapentaenoylglycerol (0.272 g. 0.00069 mol) was dissolved in methanol/chloroform/water mixture (3/3/1) (7 mL) and Dowex H+ resin (8 g) was added; the mixture was stirred at room temperature for 3 h. After filtration. the filtrate was purified by silica gel chromatography using a gradient of petroleum ether/diethyl ether to give the mono-O-eicosapentaenoylglycerol (0.15 g. 0.00038 mol. 55%). ^{1}H-NMR (600 MHz; $CD_3OD/CDCl_3$ 1/1): δ 5.49-5.35 (10H, vinyl protons), 4.23 (1H, dd, *J* = 4.4, 11.2 Hz), 4.10 (1H, dd, *J* = 6.0, 11.2 Hz), 3.95 (1H, m), 3.59 (1H, dd, *J* = 4.8, 11.2 Hz), 3.53 (1H, dd, *J* = 5.9, 11.2 Hz), 2.80–2.71 (8H, m), 2.36 (2H, t, *J* = 7.7 Hz, α-methylene), 2.16–2.01 (4H, allylic protons), 1.72 (2H, m, β-methylene), 0.86 (3H, t, *J* = 6.68 Hz). HR-ESIMS *m*/*z* 399.2474 $[M + Na]^+$.

- 1-MAG-DHA

1.2-O-isopropylidene-3-O-docosahenaenoylglycerol: 1.2-O-isopropylidene glycerol (0.325 g. 0.00077 mol) was dissolved in anhydrous dichloromethane (5 mL); docosahexaenoic acid (0.301 g. 0.00092 mol). N.N-dicyclohexylcarbodiimide (0.188 g. 0.00092 mol). and N.N-dimethylaminopyridine (0.006 g. 0.00005 mol) were added under argon and the reaction mixture was stirred overnight at room temperature; after evaporation under reduced pressure. the mixture was purified by silica gel chromatography using a gradient of petroleum ether/diethyl ether to give 1.2-O-isopropylidene-3-O-docosahexaenoylglycerol (0.300 g. 0.00068 mol. 88%).

1-mono-O-docosahexaenoylglycerol: 1.2-O-isopropylidene-3-O-docosahexaenoylglycerol (0.300 g. 0.00068 mol) was dissolved in methanol/chloroform/water mixture (3/3/1) (7 mL) and Dowex H+ resin (8 g) was added; the mixture was stirred at room temperature for 3 h. After filtration. the filtrate was purified by silica gel chromatography using a gradient of petroleum ether/diethyl ether to give the mono-O-docosohexaenoylglycerol (0.140 g. 0.00034 mol. 51%). ^{1}H-NMR (600 MHz; $CD_3OD/CDCl_3$ 1/1): δ 5.47-5.33 (12H, vinyl protons), 4.31 (1H, dd, *J* = 4.4, 11.2 Hz), 4.07 (1H, dd, *J* = 6.0, 11.2 Hz), 4.01 (1H, m), 3.59 (1H, dd, *J* = 4.8, 11.2 Hz), 3.53 (1H, dd, *J* = 5.9, 11.2 Hz), 2.80–2.71 (10H, m), 2.38 (2H, t, *J* = 7.7 Hz, α-methylene), 2.33 (2H, m, β-methylene), 2.01 (2H, allylic protons), 0.83 (3H, t, *J* = 6.68 Hz). HR-ESIMS *m*/*z* 425.2583 $[M + Na]^+$.

- 1-MAG-C16:3

1.2-O-isopropylidene-3-O-C16:3glycerol: 1.2-O-isopropylidene glycerol (0.325 g. 0.00077 mol) was dissolved in anhydrous dichloromethane (5 mL); C16:3 carboxylic acid (0.228 g. 0.00092 mol). N.N-dicyclohexylcarbodiimide (0.188 g. 0.00092 mol). and N.N-dimethylaminopyridine (0.006 g. 0.00005 mol) were added under argon and the reaction mixture was stirred overnight at room temperature; after evaporation under reduced pressure. the mixture was purified by silica gel chromatography using a gradient of petroleum ether/diethyl ether to give 1.2-O-isopropylidene-3-O-C16:3-glycerol (0.210 g. 0.00058 mol. 85%).

1-mono-O-C16:3 glycerol: 1.2-O-isopropylidene-3-O-C16:3-glycerol (0.210 g. 0.00058 mol) was dissolved in methanol/chloroform/water mixture (3/3/1) (7 mL) and Dowex H+ resin (8 g) was added; the mixture was stirred at room temperature for 3 h. After filtration. the filtrate was purified by silica gel chromatography using a gradient of petroleum ether/diethyl ether to give the mono-O-C16:3-glycerol (0.096 g. 0.00028 mol. 48%).

All synthetic products have been purified by HPLC on a Synergi Fusion RP column by using the following gradient: 80% of MeOH in H2O to 100% MeOH in 10 min. followed by 10 min 100% MeOH. A conditioning step of 10 min in 80% of MeOH was included before the successive run. Flow: 1mL/min. UV detection at λ=210 nm. Final purity of all MAGs was ≥98%. %. ^{1}H-NMR (600 MHz; $CD_3OD/CDCl_3$ 1/1): δ 5.49-5.33 (6H, vinyl protons), 4.34 (1H, dd, *J* = 4.4, 11.2 Hz), 4.09 (1H, dd, *J* = 6.0, 11.2 Hz), 4.08 (1H, m), 3.59 (1H, dd, *J* =4.8, 11.2 Hz), 3.53 (1H, dd, *J* =5.9, 11.2 Hz), 2.80–2.71 (4H, m), 2.38 (2H, t, *J* = 7.7 Hz, α-methylene), 2.16-2.01 (4H, allylic protons), 1.76 (2H, m, β-methylene), 1.36–1.24 (4H, aliphatic protons), 0.82 (3H, t, *J* = 6.68 Hz). HR-ESIMS *m*/*z* 347.2123 $[M + Na]^+$.

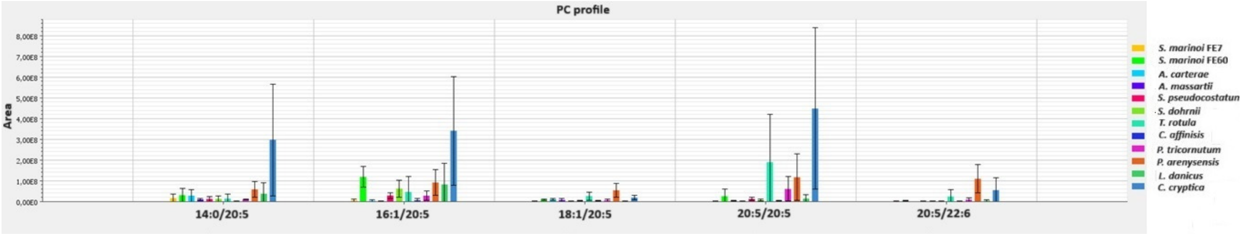

Figure S1. PC composition in the selected microalgal species. Data are reported as mean peak Area ± SD (n=3).

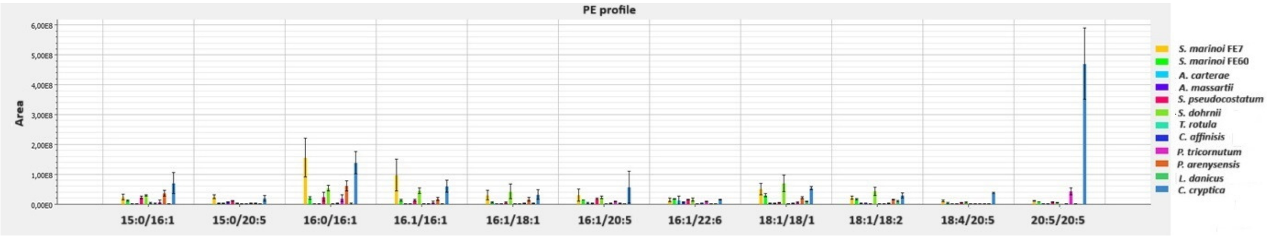

Figure S2. PE composition in the selected microalgal species. Data are reported as mean peak Area ± SD (n=3).

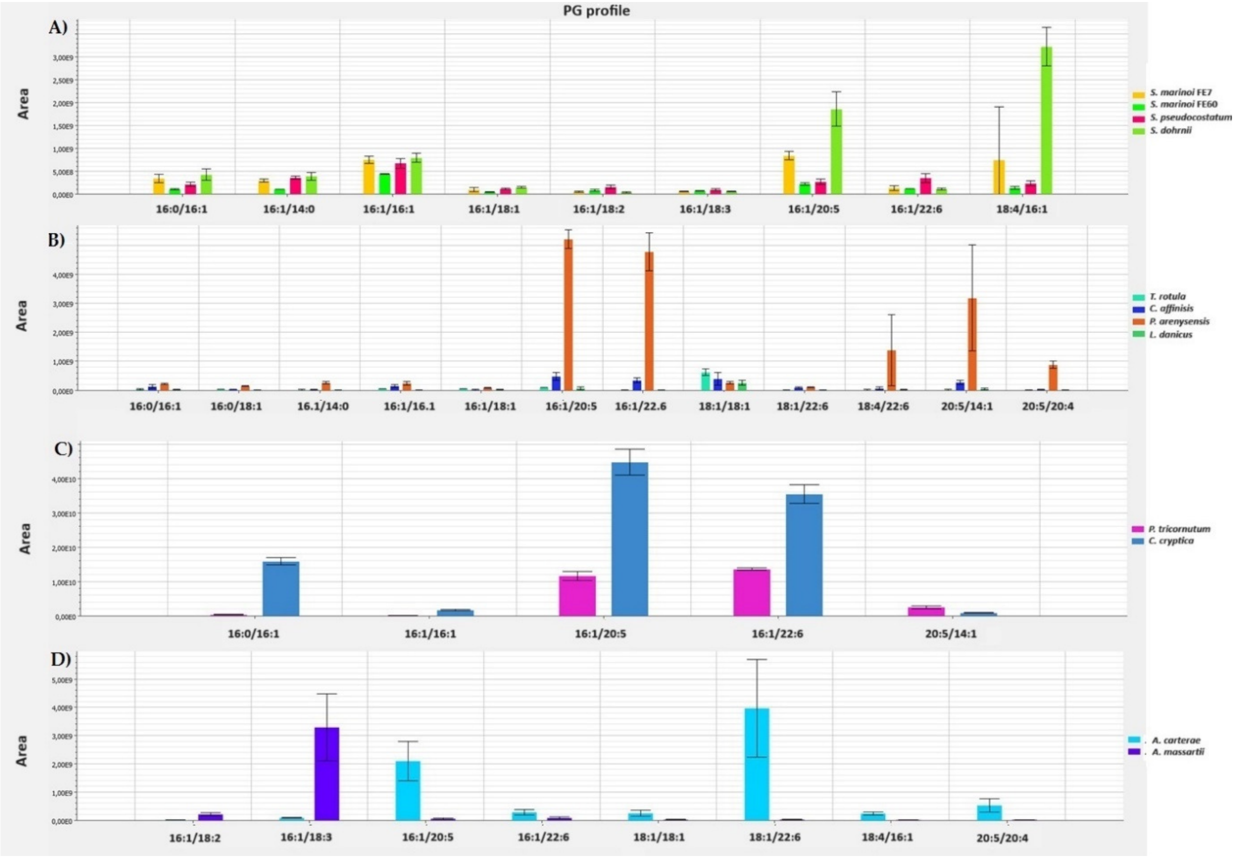

Figure S3. PG composition in the selected microalgal species. Distribution of PG in **(A)** four *Skeletonema* spp. **(B)** other colonial and **(C)** non colonial diatoms **(D)** and the two *Amphidinium* species. Data are reported as mean peak Area ± SD (n=3).

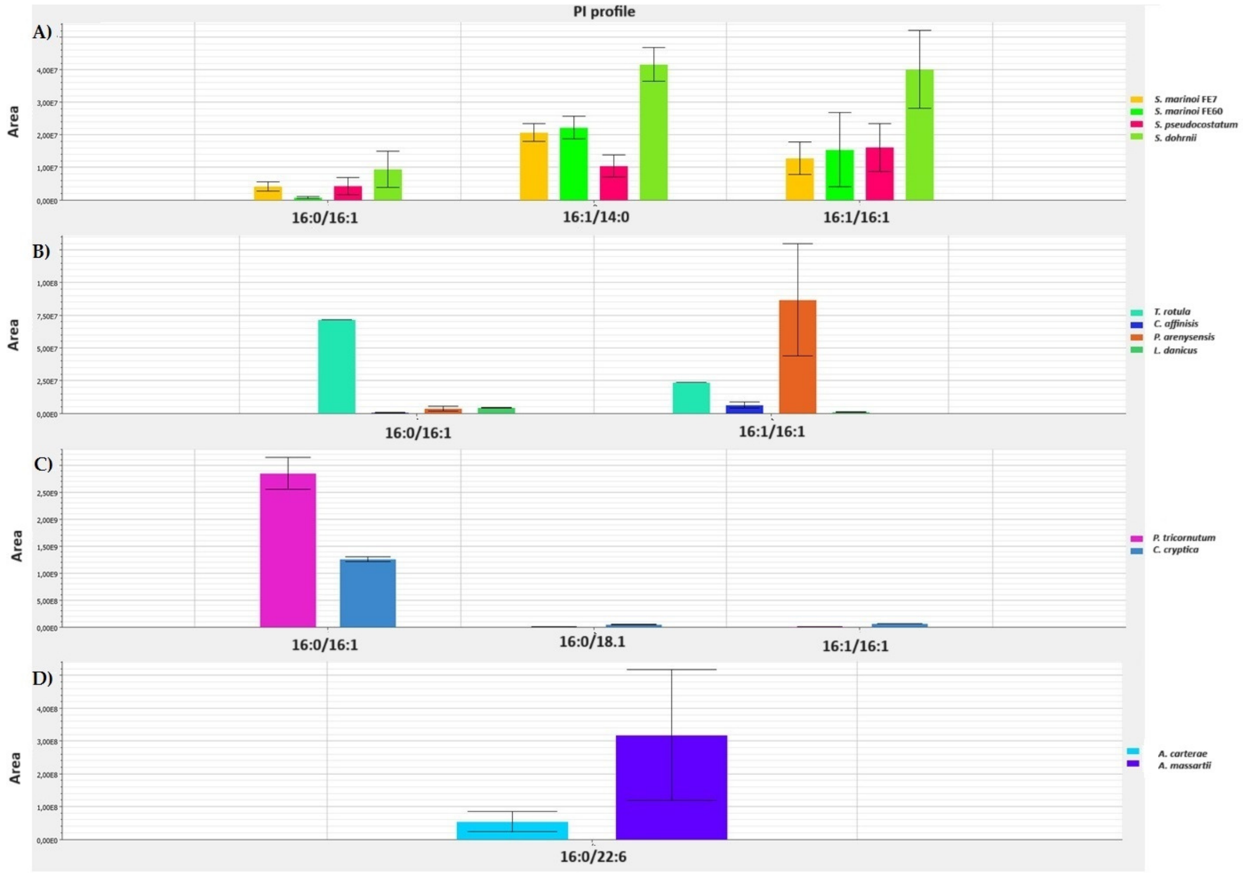

Figure S4. PI composition in the selected microalgal species. Distribution of PI in **(A)** four *Skeletonema* spp. (**B)** other colonial and (**C**) non colonial diatoms (**D**) and the two *Amphidinium* species. Data are reported as mean peak Area ± SD (n=3).

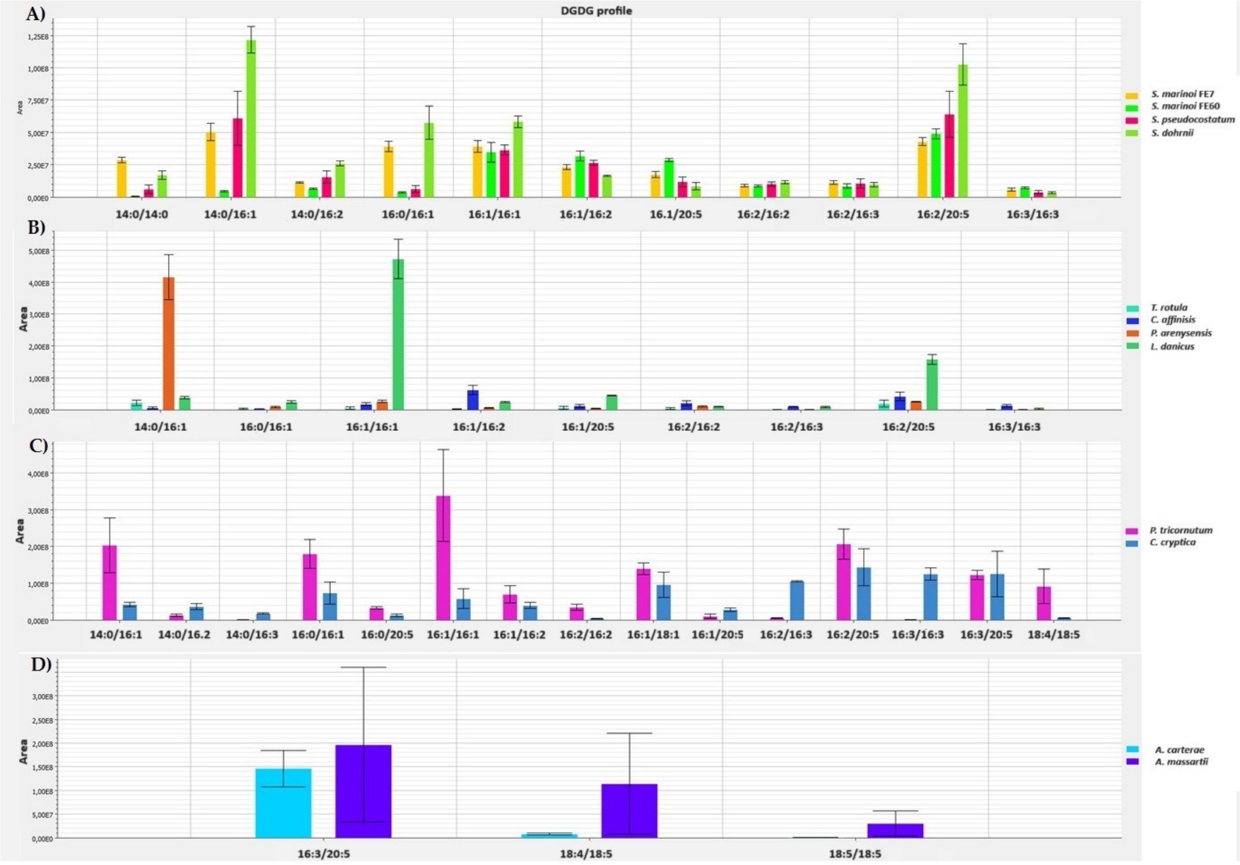

Figure S5. DGDG composition in the selected microalgal species. Distribution of DGDG in **(A)** four *Skeletonema* spp. (**B)** other colonial and (**C**) non colonial diatoms (**D**) and the two *Amphidinium* species. Data are reported as mean peak Area ± SD (n=3).

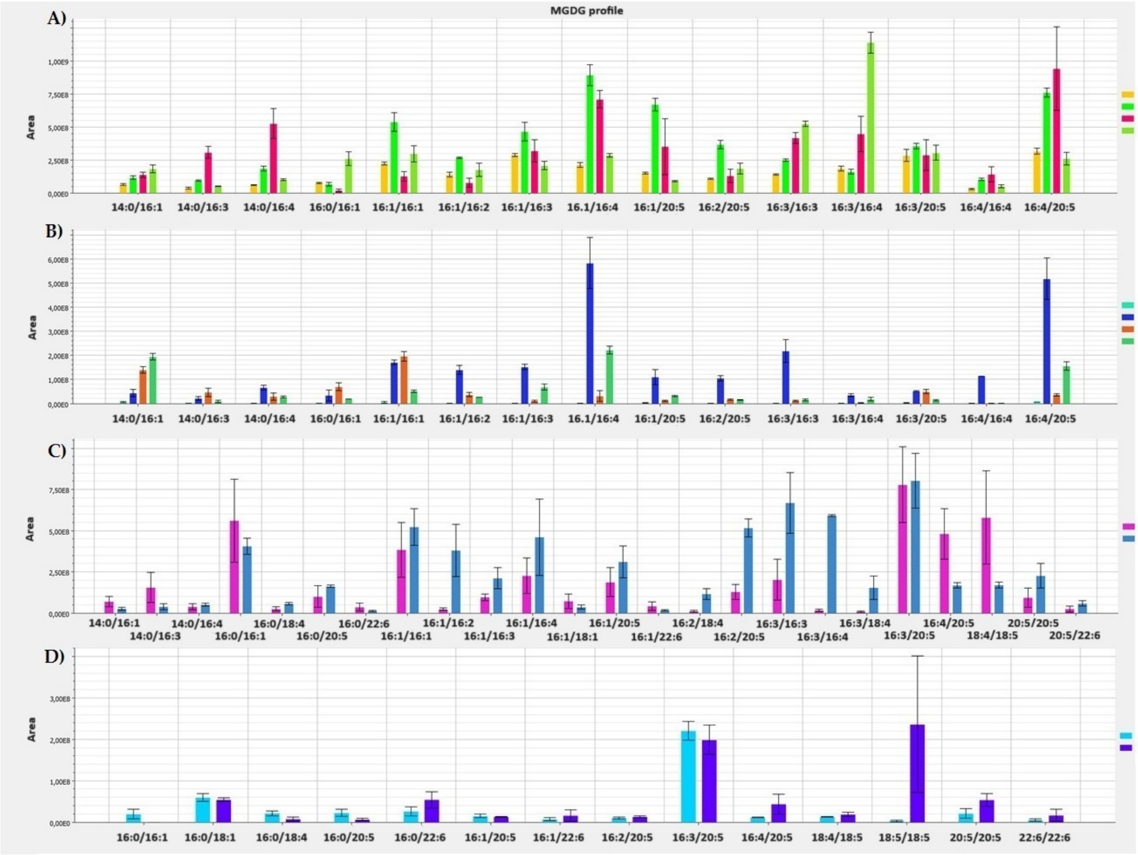

Figure S6. MGDG composition in the selected microalgal species. Distribution of MGDG in **(A)** four *Skeletonema* spp. (**B)** other colonial and (**C**) non colonial diatoms (**D**) and the two *Amphidinium* species. Data are reported as mean peak Area ± SD (n=3).

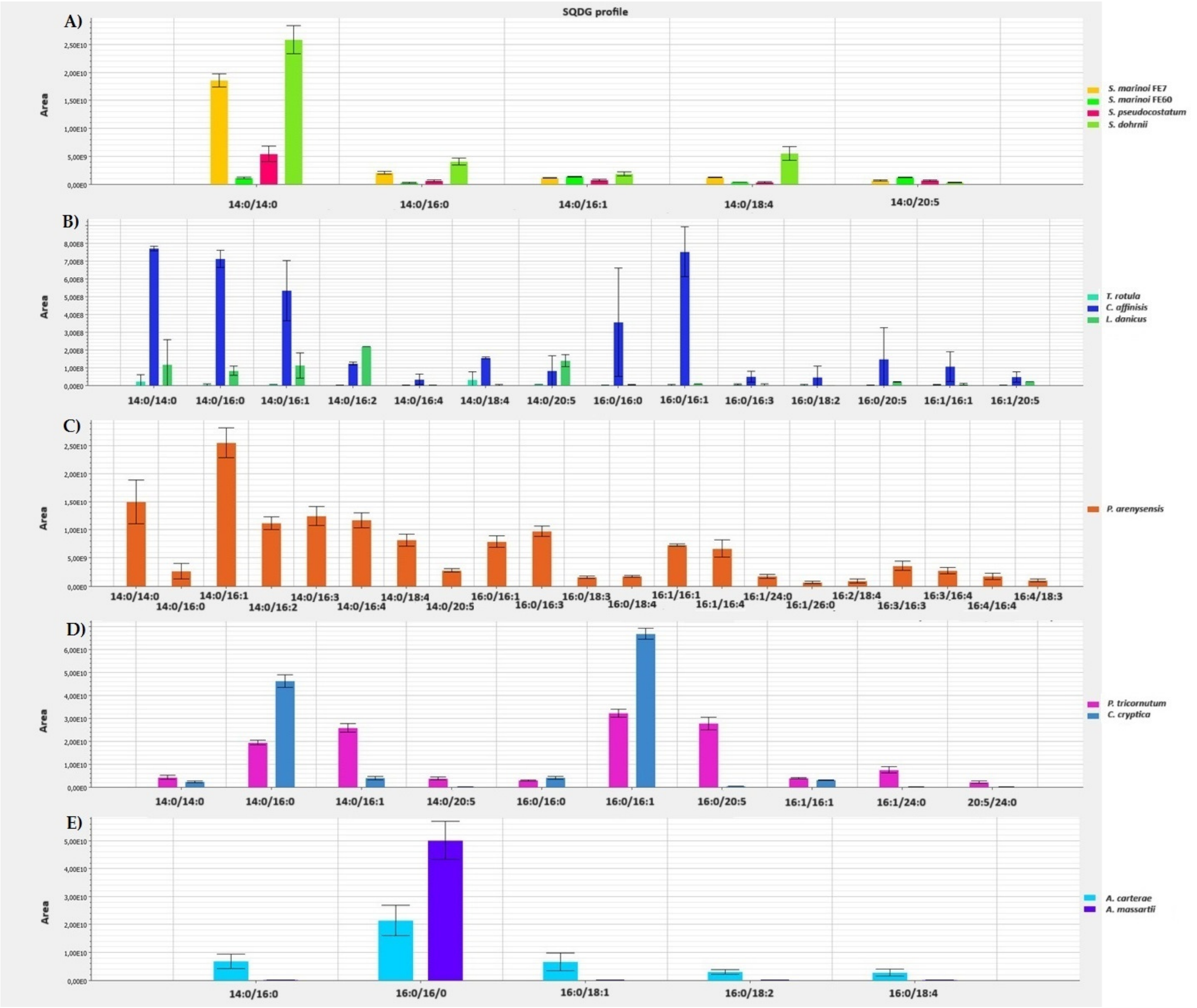

Figure S7. SQDG composition in the selected microalgal species. Distribution of SQDG in **(A)** four *Skeletonema* spp. **(B-C)** colonial and (**D**) non colonial diatoms (**E**) and the two *Amphidinium* species. Data are reported as mean peak Area ± SD (n=3).

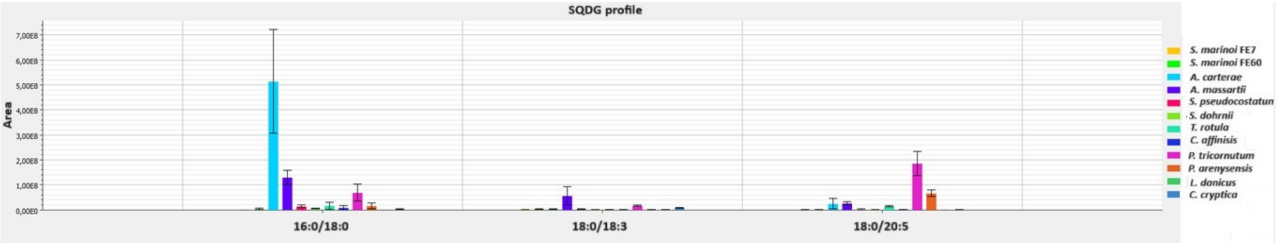

Figure S8. SQDG containing C18:0 fatty acid in the selected microalgal species. Data are reported as mean peak Area ± SD (n=3)..

Table S1. Abundance of main MAG quantified by LC-MS analysis and expressed as µg/mg of DW. Means ± SD (n=3).

	Skeletonema marinoi FE7		*Skeletonema marinoi* FE60		*Skeletonema dohrnii*		*Skeletonema pseudocostatum*		*Chaetoceros affinis*		*Thalassiosira rotula*		*Pseudo-nitzschia arenysensis*		*Leptocylindrus danicus*		*Pheodactylum tricornutum*		*Cyclotella cryptica*		*Amphidinium massartii*		*Amphidinium carterae*	
	**µg/mg DW**	**SD**	**µg/mg DW**	**SD**	**µg/mg DW**	**SD**	**µg/mg DW**	**SD**	**µg/mg DW**	**SD**	**µg/mg DW**	**SD**	**µg/mg DW**	**SD**	**µg/mg DW**	**SD**	**µg/mg DW**	**SD**	**µg/mg DW**	**SD**	**µg/mg DW**	**SD**	**µg/mg DW**	**SD**
**MAG-C14:0**	0.0872	0.0231	-	-	0.0782	0.0216	0.0495	0.0323	0.0083	0.0045	-	-	-	-	-	-	-	-	-	-	-	-	0.0016	0.0018
**MAG-C16:0**	1.2962	0.1803	0.1196	0.0197	0.1952	0.1305	0.1777	0.0219	0.4680	0.0260	0.2966	0.1062	1.1380	0.1267	0.8836	0.0676	0.2469	0.0626	1.1001	0.1575	0.0309	0.0225	0.7906	0.2187
**MAG-C16:1**	1.2463	0.1049	0.1388	0.0342	1.2294	0.1801	1.0203	0.2201	1.2679	0.1222	0.6093	0.2439	-	-	-	-	0.1387	0.0820	0.0626	0.0225	-	-	-	-
**MAG-C16:2**	0.1352	0.0161	0.0047	0.0022	0.0270	0.0089	0.0860	0.0239	0.2169	0.0097	0.0283	0.0290	-	-	-	-	-	-	-	-	-	-	-	-
**MAG-C16:3**	0.1995	0.0427	0.0951	0.0085	0.0270	0.0089	0.2480	0.0355	0.0324	0.0057	0.0377	0.0146	0.0142	0.0037	-	-	0.0066	0.0036	-	-	-	-	-	-
**MAG-C16:4**	0.0541	0.0039	0.0272	0.0109	0.0884	0.0137	0.2516	0.1243	0.1386	0.0133	0.0202	0.1212	-	-	-	-	-	-	-	-	-	-	-	-
**MAG-C18:0**	1.0778	0.1212	0.1106	0.0143	0.0750	0.0340	0.2046	0.0219	0.0964	0.0208	0.0136	0.0023	0.7872	0.0987	0.5420	0.0849	0.0779	0.0168	0.8062	0.0492	0.1717	0.0493	0.1859	0.0534
**MAG-C18:1**	-	-	-	-	-	-	-	-	0.0220	0.0067	-	-	-	-	-	-	-	-	-	-	-	-	0.3308	0.1211
**MAG-C18:2**	0.0179	0.0039	-	-	0.0096	0.0014	0.0117	0.0019	0.0149	0.0041	0.0160	0.0186	-	-	-	-	-	-	-	-	-	-	0.0147	0.0033
**MAG-C18:3**	0.0109	0.0020	0.0028	0.0007	0.0111	0.0026	0.0128	0.0042	0.0269	0.0062	0.0220	0.0104	0.0973	0.0352	0.0009	0.0003	0.0090	0.0043	-	-	-	-	0.0105	0.0015
**MAG-C18:4**	0.0279	0.0044	0.0005	0.0002	0.0451	0.0093	0.0180	0.0075	0.0458	0.0064	0.1269	0.0135	-	-			0.0038	0.0032	0.0018	0.0015	0.0760	0.0187	0.1713	0.0700
**MAG-C20:0**	0.0352	0.0032	-	-	-	-	-	-	-	-	-	-	-	-	0.0093	0.0012	-	-	0.0206	0.0104	-	-	-	-
**MAG-C20:3**	0.0037	0.0036	0.0006	0.0003	0.0006	0.0004	0.0017	0.0004	0.0003	0.0004	-	-	0.0076	0.0029	0.0028	0.0015	0.0007	0.0001	0.0037	0.0017	0.0014	0.0005	-	-
**MAG-C20:4**	0.0053	0.0025	-	-	-	-	0.0006	0.0005	0.0144	0.0036	0.0034	0.0042	-	-	-	-	-	-	-	-	0.0108	0.0020	-	-
**MAG-C20:5**	0.5707	0.0647	0.1615	0.0272	0.5745	0.1458	0.4262	0.0808	0.4626	0.0885	0.3282	0.1306	-	-	-	-	0.0289	0.0155	0.0661	0.0254	-	-	0.1901	0.0838
**MAG-C22:6**	0.0864	0.0185	0.0801	0.0126	0.0521	0.0083	0.2797	0.0192	0.1194	0.0258	-	-	-	-	-	-	-	-	-	-	0.0654	0.0221	0.3314	0.1430
**Total amount**	**4.85**		0.74		**2.41**		**2.79**		**2.93**		1.50		**2.04**		1.44		0.51		**2.06**		0.36		**2.03**	

Table S2. Abundance of main MAG quantified by LC-MS analysis and expressed as µg/mg lipid extract. Means ± SD (n=3).

	Skeletonema marinoi FE7		*Skeletonema marinoi* FE60		*Skeletonema dohrnii*		*Skeletonema pseudocostatum*		*Chaetoceros affinis*		*Thalassiosira rotula*		*Pseudo-nitzschia arenysensis*		*Leptocylindrus danicus*		*Pheodactylum tricornutum*		*Cyclotella cryptica*		*Amphidinium massartii*		*Amphidinium carterae*	
	µg/mg	SD	µg/mg	SD	µg/mg	SD	µg/mg	SD	µg/mg	SD	µg/mg	SD	µg/mg	SD	µg/mg	SD	µg/mg	SD	µg/mg	SD	µg/mg	SD	µg/mg	SD
**MAG-C14:0**	0.70	0.09	-	-	0.71	0.19	0.32	0.19	0.05	0.03	-	-	-	-	-	-	-	-	-	-	-	-	-	-
**MAG-C16:0**	10.32	1.56	1.06	0.19	1.70	1.08	1.25	0.04	2.77	0.30	1.13	0.37	9.50	0.92	5.21	0.24	1.49	0.59	4.92	0.63	0.13	0.08	3.96	1.03
**MAG-C16:1**	11.22	0.68	1.24	0.33	10.90	1.61	7.11	0.86	7.84	0.98	2.43	1.21	-	-	-	-	0.90	0.66	0.28	0.10	-	-	-	-
**MAG-C16:2**	1.12	0.16	0.04	0.02	0.24	0.08	0.61	0.18	1.29	0.16	0.12	0.13	<0.01	-	<0.01	-	-	-	-	-	-	-	-	-
**MAG-C16:3**	1.62	0.16	0.85	0.07	3.58	0.79	1.75	0.20	0.19	0.04	0.08	0.17	0.12	0.02	<0.01	-	0.04	0.02	0.01	0.00	-	-	-	-
**MAG-C16:4**	0.49	0.03	0.24	0.08	0.78	0.11	1.69	0.59	0.82	0.11	0.19	0.13	<0.01	-	<0.01	-	-	-	-	-	-	-	-	-
**MAG-C18:0**	8.95	2.81	0.99	0.15	0.68	0.29	1.47	0.32	0.57	0.12	0.05	0.01	6.56	0.63	3.22	0.65	0.45	0.13	3.61	0.23	0.77	0.24	0.92	0.23
**MAG-C18:1**	-	-	-	-	-	-	-	-	0.13	0.05	-	-	-	-	-	-	-	-	-	-	-	-	1.64	0.51
**MAG-C18:2**	0.14	0.02	-	-	0.08	0.01	0.08	0.02	0.09	0.02	<0.01	-	-	-	-	-	-	-	-	-	-	-	0.07	0.02
**MAG-C18:3**	0.10	0.02	0.02	0.01	0.10	0.02	0.09	0.02	0.16	0.05	0.09	0.04	0.80	0.24	<0.01	-	0.06	0.03	-	-	-	-	0.05	0.01
**MAG-C18:4**	0.26	0.04	<0.01	-	0.41	0.07	0.12	0.03	0.27	0.06	0.53	0.13	-	-	-	-	0.02	0.02	0.01	0.01	0.34	0.13	0.85	0.41
**MAG-C20:0**	0.32	0.02	-	-	-	-	-	-	-	-	-	-	-	-	0.05	0.01	-	-	0.09	0.05	-	-	-	-
**MAG-C20:4**	0.04	0.01	-	-	-	-	<0.01	-	0.09	0.03	0.01	0.02	-	-	-	-	-	-	-	-	0.04	0.01	-	-
**MAG-C20:5**	5.35	0.66	1.43	0.24	5.17	1.16	2.98	0.37	2.70	0.42	1.28	0.55	-	-	-	-	0.19	0.10	0.30	0.11	-	-	0.96	0.46
**MAG-C22:6**	0.65	0.13	0.72	0.14	0.46	0.20	1.98	0.24	0.70	0.15	<0.01	-	-	-	-	-	-	-	-	-	0.29	0.09	1.65	0.74
**Total amount**	**41.28**		6.59		**24.81**		**19.45**		**17.67**		5.91		**16.98**		8.48		3.15		9.22		1.57		10.10	

Table S3. Levels of main fatty acids. identified and quantified by GC-MS analysis as methyl ester derivatives (FAME). Values are expressed as µg/mg DW. Means ± SD (n=3).

	Skeletonema marinoi FE7		*Skeletonema marinoi* FE60		*Skeletonema dohrnii*		*Skeletonema pseudocostatum*		*Chaetoceros affinis*		*Thalassiosira rotula*		*Pseudo-nitzschia arenysensis*		*Leptocylindrus danicus*		*Pheodactylum tricornutum*		*Cyclotella cryptica*		*Amphidinium massartii*		*Amphidinium carterae*	
**FAME**	**µg/mg DW**	**SD**	**µg/mg DW**	**SD**	**µg/mg DW**	**SD**	**µg/mg DW**	**SD**	**µg/mg DW**	**SD**	**µg/mg DW**	**SD**	**µg/mg DW**	**SD**	**µg/mg DW**	**SD**	**µg/mg DW**	**SD**	**µg/mg DW**	**SD**	**µg/mg DW**	**SD**	**µg/mg DW**	**SD**
**C14:0**	2.87	0.14	2.47	0.14	2.69	0.06	4.94	0.15	12.32	0.30	6.17	0.29	5.20	0.63	7.09	0.61	3.64	0.20	5.90	0.28	0.58	0.17	-	
**C15:0**	0.16	0.01	0.09	0.01	0.16	0.00	0.19	0.02	0.35	0.02	0.27	0.02	0.23	0.05	0.44	0.11	0.84	0.06	0.24	0.03	0.04	0.01	-	
**C16:4**	1.46	0.07	1.75	0.24	1.74	0.07	3.02	0.19	0.68	0.15	0.90	0.23	2.28	0.42	2.66	0.12	-		2.12	0.35	-		-	
**C16:3**	3.09	0.17	1.71	0.19	3.45	0.06	2.52	0.13	0.40	0.05	0.28	0.03	3.71	0.74	3.51	0.13	17.52	1.35	0.57	0.11	-		-	
**C16:1**	11.75	0.18	9.70	0.58	9.61	0.12	7.51	0.33	19.02	0.66	15.68	0.61	46.52	5.87	19.10	1.36	39.71	2.10	14.28	0.75	0.12	0.02	0.01	0.01
**C16:2**	1.74	0.04	1.75	0.19	1.64	0.13	1.14	0.04	0.67	0.03	1.81	0.13	2.28	0.51	3.12	0.37	4.05	0.23	3.98	0.23	-		-	
**C16:0**	2.36	0.05	1.95	0.12	2.54	0.05	1.65	0.06	5.25	0.27	7.33	0.26	25.07	2.99	4.61	0.68	23.25	1.30	4.79	0.49	10.61	2.03	11.05	0.84
**C18:3 ω6**	0.08	0.01	0.06	0.01	0.09	0.00	0.14	0.01	-		-		-		-		0.18	0.02	0.09	0.01	-		-	
**C18:3 ω3**	0.08	0.02	0.13	0.01	-		0.04	0.00	0.73	0.03	0.18	0.04	0.73	0.09	0.23	0.02	0.18	0.03	0.24	0.03	0.04	0.01	0.04	0.04
**C18:4 ω3**	1.36	0.02	0.65	0.12	1.16	0.03	0.44	0.04	0.98	0.22	0.19	0.04	1.84	0.15	0.45	0.07	4.91	0.20	0.37	0.09	5.08	0.36	12.07	1.13
**C18:2 ω6**	0.27	0.01	0.36	0.05	0.41	0.00	0.20	0.02	0.35	0.01	0.13	0.01	3.23	0.59	1.13	0.07	0.24	0.01	2.26	0.29	0.36	0.05	0.24	0.01
**C18:1 ω9**	0.14	0.00	0.23	0.01	0.24	0.00	0.58	0.03	1.27	0.03	0.33	0.04	4.87	0.64	3.32	0.09	0.28	0.02	2.43	0.75	3.13	1.06	0.66	0.04
**C18:1 ω7**	0.41	0.05	0.36	0.05	0.59	0.02	0.08	0.01	0.93	0.06	0.96	0.05	0.74	0.10	0.97	0.19	0.74	0.07	0.38	0.05	0.35	0.06	0.01	0.01
**C18:2 ωx**	0.47	0.10	0.19	0.07	0.76	0.10	1.04	0.08	1.05	0.02	0.83	0.07	0.59	0.14	2.53	0.28	2.53	0.20	2.25	0.06	1.64	0.20	2.11	0.07
**C18:0**	0.22	0.03	0.25	0.04	0.20	0.01	0.19	0.01	0.34	0.01	0.42	0.02	0.82	0.07	0.54	0.08	0.67	0.08	0.42	0.05	1.00	0.12	1.98	0.15
**AA**	-		0.03	0.01	-		-		0.05	0.01	0.02	0.00	0.48	0.10	0.13	0.03	-		0.20	0.10	-		0.27	0.04
**EPA**	7.48	0.04	6.71	1.27	6.99	0.04	7.69	0.55	2.16	0.48	4.77	1.19	35.13	5.04	5.37	0.98	24.06	1.14	6.71	1.07	3.26	0.23	9.55	0.82
**C21:0**	-		-		-		-		0.03	0.00	-		-		0.22	0.02	-		-		1.73	0.19	3.83	0.35
**DHA**	0.99	0.01	0.92	0.21	1.12	0.01	1.12	0.10	0.14	0.05	0.22	0.07	1.83	0.26	0.30	0.08	3.25	0.14	1.42	0.20	2.41	0.22	11.60	0.71
**C22:0**	-		-		-		-		0.02	0.00	-		0.12	0.02	1.07	0.25	-		-		0.36	0.04	1.00	0.09
**C24:0**	-		-		-		-		-		0.11	0.01	3.10	0.51	3.07	0.67	0.03	0.00	-		0.75	0.09	0.52	0.05
**Total**	34.90		29.29		33.41		32.51		46.75		40.60		138.76		59.86		126.06		48.66		31.45		54.94	
